# Drug-induced cholestasis assay in primary hepatocytes

**DOI:** 10.1016/j.mex.2020.101080

**Published:** 2020-09-25

**Authors:** Pieter Van Brantegem, Sagnik Chatterjee, Tom De Bruyn, Pieter Annaert, Neel Deferm

**Affiliations:** a KU Leuven Department of Pharmaceutical and Pharmacological Sciences, Leuven, Belgium; b Pharmaceutical Candidate Optimization, Biocon, Bristol-Myers Squibb R& D Center (BBRC), Syngene International Ltd., Bangalore, India; c Department of Drug Metabolism and Pharmacokinetics, Genentech Inc, South San Francisco, CA, USA

**Keywords:** Drug-induced liver injury, Drug-induced cholestasis, *In vitro* toxicity, Drug candidate selection, Primary hepatocytes, Drug safety evaluation

## Abstract

Drug-induced cholestasis (DIC) is a major cause of clinical failure of drug candidates. Numerous patients worldwide are affected when exposed to marketed drugs exhibiting a DIC signature. Prospective identification of DIC during early compound development remains challenging. Here we describe the optimized *in vitro* procedure for early assessment and prediction of an increased DIC risk. Our method is based on three principles:

• Exposure of primary human hepatocyte cultures to test compounds in the absence and presence of a physiologically relevant mixture of endogenous bile salts.
• Rapid and quantitative assessment of the influence of concomitant bile salt exposure on hepatocyte functionality and integrity after 24 h or 48 h of incubation.
• Translation of the *in vitro* result, expressed as a DIC index (DICI) value, into an *in vivo* safety margin.

Using our historical control data, a new (data driven) DICI cut-off value of 0.78 was established for discerning cholestatic and non-cholestatic compounds. Our DIC assay protocol was further improved by now relying on the principle of the no observable adverse effect level (NOAEL) for determining the highest test compound concentration corresponding to a DICI  ≥  0.78. Predicted safety margin values were subsequently calculated for compounds displaying hepatotoxic and/or cholestatic effects in patients, thus enabling evaluation of the performance of our DIC assay. Of note, this assay can be extended to explore the role of drug metabolites in precipitating DIC.

## Graphical abstract

**Figure:**
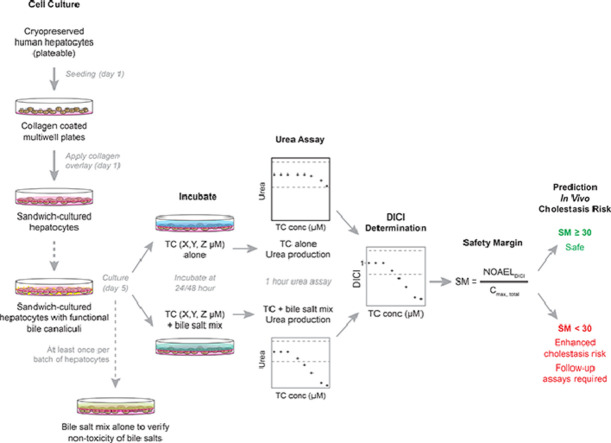

Specifications Table

**Table:** 

Subject Area	Pharmacology, Toxicology and Pharmaceutical Science
More specific subject area	In vitro drug safety evaluation
Method name	DIC assay
Name and reference of original method	1. S. Chatterjee, L. Richert, P. Augustijns, P. Annaert. Hepatocyte-based in vitro model for assessment of drug-induced cholestasis. Toxicology and applied pharmacology 274, 124-136 (2014). 2. M. Oorts, A. Baze, P. Bachellier, B. Heyd, T. Zacharias, P. Annaert, L. Richert. Drug-induced cholestasis risk assessment in sandwich-cultured human hepatocytes. Toxicol In Vitro 34, 179-186 (2016).
Resource availability	N.A.

## General description of the method

Drug-induced cholestasis (DIC) is a major cause of attrition during drug development and post-marketing withdrawal [1]. Early detection of DIC is crucial for avoiding financial losses and improving drug safety. Preclinical assays for DIC detection often rely on evaluation of the test compound towards individual cellular targets such as their inhibitory potential towards the bile salt export pump (BSEP) by using BSEP membrane vesicles [2,3]. However, the underlying mechanisms of DIC development are more complex involving multiple other transporters and nuclear receptors [4]. A more comprehensive *in vitro* model such as sandwich-cultured human hepatocytes (SCHH) are more suited due to the presence of metabolizing enzymes and transporters relevant to bile salt disposition [5]. The assay described here relies on changes in the ability of SCHH to produce urea from ammonia in the absence and presence of a (patho-)physiologically relevant bile salt mixture. Ammonia is detoxified in the hepatocyte by the urea cycle to form non-toxic urea. Measuring changes in urea production allows for the quantitative evaluation of the hepatocyte's biochemical and functional integrity. The colorimetric quantitation of urea formation is based on the reaction between urea and diacetylmonoxime.

By determining the ratio of urea formation after incubation with test compound in absence and in presence of the bile salt mixture, the DIC index (DICI) value can be calculated. Based on previously published protocols, a DICI value lower than 0.80 was used to flag a test compound at that specific concentration for an increased *in vitro* DIC risk [6], [7], [8]. In addition, following evaluation of a range of test compound concentrations, a safety margin (SM) could be estimated by dividing the lowest concentration with DICI < 0.80 (cf. a lowest adverse effect level, LOAEL) *in vitro,* by the reported total peak plasma concentration (*C*_max,total_) of the test compound in humans. It was previously established that a compound with a SM below 30 can be considered to display increased risk for causing cholestasis in the clinic [7].

The previously proposed DICI cut-off value of 0.80 was empirically set to take into account the intrinsic variability of baseline urea formation in the sandwich-culture system. We presently introduce two modifications to the previously established DIC assay. First, we now calculate the DICI value based on the no observed adverse effect level (NOAEL) instead of using the LOAEL. The NOAEL is a widely used approach for safety evaluations supported by regulatory agencies [9], and is more conservative as compared to the LOAEL. Second, we provided a statistical perspective around the DICI cut-off value, considering all the experiments carried out by our group so far. The 90% confidence interval (CI) of the DICI values for the control conditions (*i.e.* incubation with 0.2% dimethylsulfoxide (DMSO) or the bile salt mixture alone) throughout all our previously performed human hepatocyte-based experiments, was determined. Whenever a test compound's DICI value falls within the CI of the historical controls, the corresponding concentration is considered ‘safe’ in terms of cholestasis. In practice, a compound tests ‘positive’ for (*in vitro*) DIC at a given concentration, when the obtained DICI value falls below the lower bound of the 90% CI of the controls, *i.e.* 0.78.

The SM was set at 30 based on the SM determined by Yao et al. [10]. We reassessed our previously generated data and verified the SM for prediction of DIC *in vivo* using our assay after 48 h incubation with receiver operating characteristic (ROC) analysis.

## Experimental procedure

**Fig. 1:**
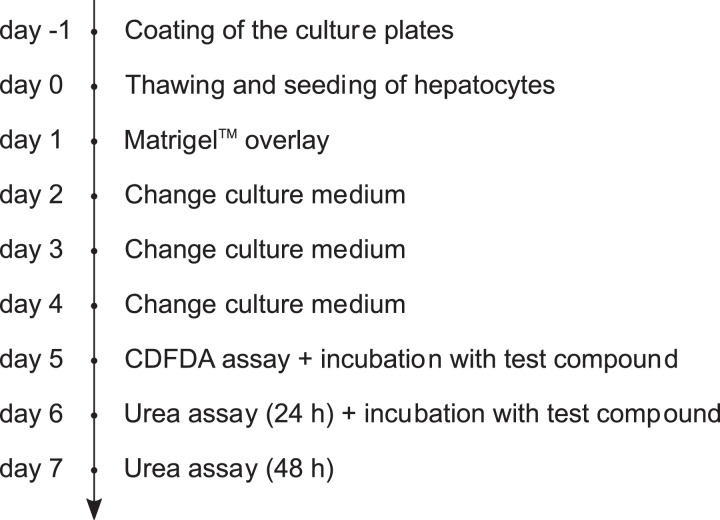
Timeline of the experimental part of the DIC assay.

A timeline of the different steps of the experimental part of the assay can be found in Fig. 1.

All actions should be performed under a laminar airflow cabinet.

## Materials

Materials described here are general equipment or materials necessary throughout the whole experiment. Specific reagents are described in each section.

• Purified water.
• DMSO.
• 0.2 µm filters for sterile filtration.
• Laminar airflow cabinet.
• Falcon tubes.
• Thermostat water baths at 37°C and 85°C.
• Humidified incubator with 5% CO_2_.
• Centrifuge.
• Aspirator.
• Fridge.
• Fluorescence microscope.
• Absorbance plate reader.
• Computer with data analysis software such as MS Excel.

### Coating of 48-well cell culture plates with rigid collagen

Coating of the plates should preferably be performed on the day before seeding.

#### Materials

• Sterile acetic acid stock solution of 0.02 M in purified water, filtered through a 0.2 µm filter in the laminar airflow cabinet.
• Rat tail collagen stock solution of 3.5 mg/mL.
• Sterile 48-well culture plates.

#### Protocol

1. Prepare a fresh 50 µg/mL rat tail collagen solution by diluting the 3.5 mg/mL stock solution with 0.02 M acetic acid solution.
2. Add 250 µL of this collagen solution to each well of the 48-well plate and incubate at 37°C in a humidified incubator with 5% CO_2_ for at least 1 h and preferably overnight.

### Thawing of cryopreserved human hepatocytes

#### Materials

• Freshly prepared thawing medium: Dulbecco's Modified Eagle's Medium supplemented with 10% (v/v) fetal bovine serum (FBS), 1 µM dexamethasone, 4 µg/mL insulin, 100 IU/mL penicillin, 100 µg/mL streptomycin and 2 mM L-glutamine, filtered through a 0.2 µm filter and prewarmed in a water bath at 37°C.
• Freshly prepared seeding medium: Williams’ E medium supplemented with 10% (v/v) FBS, 1 µM dexamethasone, 4 µg/mL insulin, 100 IU/mL penicillin, 100 µg/mL streptomycin and 2 mM L-glutamine, filtered through a 0.2 µm filter and prewarmed in a water bath at 37°C.
• Isotonic 90% PercollⓇ prepared under sterile conditions by mixing 9 parts PercollⓇ with 1 part phosphate buffered saline (PBS).
• Trypan blue.
• Counting chamber.

#### Procedure

1. Add 16 mL of the 90% PercollⓇ solution to a 50 mL falcon tube and add 25 mL thawing medium. Prewarm in a water bath at 37°C.
2. Carefully remove the cryovials from the liquid nitrogen tank or freezer, with specific attention to safety precautions (especially in terms of wearing personal protective equipment).
3. Immediately warm the vials in a water bath at 37°C. Swirl gently until the ice crystals are completely melted.
4. Open the vials in the laminar airflow cabinet and transfer the content of the cryovial to the mixture of 90% PercollⓇ and thawing medium.
5. Rinse the cryovial with 1 mL thawing medium and adjust the volume of the PercollⓇ-thawing medium mixture to 50 mL.
6. Close the lid of the falcon tube and resuspend the cells by gently inverting the tube 2–3 times.
7. Centrifuge the cell suspension at room temperature for 20 min at 168  ×  *g*.
8. Aspirate the supernatant and subsequently loosen the pellet.
  Note: it is important to first loosen the pellet. If medium is added first, the pellet will form clumps and will be difficult to resuspend.
9. Add 20 mL of prewarmed thawing medium and invert 2–3 times.
10. Centrifuge the cell suspension at room temperature for 5 min at 100  ×  *g*.
11. Aspirate the medium and resuspend the pellet as described in step 8.
12. Add 2–5 mL seeding medium (approximately 3 mL per 10 million cells).
13. Determine the total cell count and viability with the Trypan blue exclusion method. The viability should be at least 70%.

### Seeding of human hepatocytes

#### Materials

• PBS.
• Freshly prepared seeding medium, filtered through a 0.2 µm filter and prewarmed in a water bath at 37°C.

#### Procedure

1. Aspirate the collagen solution from the wells and wash three times with 250 µL prewarmed sterile PBS solution.
2. Dilute the cell suspension to 1 million cells/mL with seeding medium.
3. Transfer 250 µL of the cell suspension to each well and gently move the plate orthogonally to evenly distribute the cells over the well.
  Note 1: It is advisable to start with seeding one well and verifying the density under the microscope before seeding all wells. Dilute the cell suspension further or seed higher volumes in case of over-seeding or under-seeding, respectively.
  Note 2: Do not swirl the content of the plate with circular movements as this might cause the cells to be unevenly distributed over the surface of the wells.
4. Incubate the cells at 37°C in a humidified incubator with 5% CO_2_ for 24 h.
5. Start thawing Matrigel™ overnight in the fridge.

### Matrigel™ overlay and culture of human hepatocytes in sandwich configuration

#### Materials

• Culture medium: Williams’ E medium supplemented with 1% (v/v) ITS+™ Premix, 0.1 µM dexamethasone, 100 IU/mL penicillin, 100 µg/mL streptomycin and 2 mM L-glutamine, filtered through a 0.2 µm filter and cooled down in the fridge.
• Matrigel™ 0.25 mg/mL solution, prepared by diluting the commercial Matrigel™ in cold culture medium.
  Note: keep this solution cold as warming will cause the Matrigel™ to solidify.

#### Procedure

1. Shake the plate rigorously and aspirate the unadhered cells.
2. Add 250 µL of the diluted Matrigel™ solution and incubate the cells at 37°C in a humidified incubator with 5% CO_2_ for 24 h.
3. Replace the medium daily with prewarmed culture medium.

### Verification of the presence of bile canaliculi

The presence of bile canaliculi is confirmed with 5(6)-carboxy-2′,7′-dichlorofluorescein diacetate (CDFDA). CDFDA is a fluorogenic compound that passively diffuses through the cell membrane of the hepatocytes [11] and is subsequently converted to the fluorescent 5(6)-carboxy-2′,7′-dichlorofluorescein (CDF) by intracellular esterases. CDF is actively excreted into the bile canaliculi by the multidrug resistance-associated protein 2 (MRP2) [12]. This assay is typically performed one day in advance of the actual experiment. Make sure to reserve at least one extra well to perform the assay. If no bile canaliculi are observed, replace the medium with culture medium and repeat the assay on the next day.

#### Materials

• Standard buffer: Hanks’ balanced salt solution (HBSS) with 10  mM 4-(2-hydroxyethyl)-1-piperazineethanesulfonic acid (HEPES), adjusted to pH 7.4, filtered through a 0.2 µm filter and prewarmed in a water bath at 37°C.
• CDFDA 4 µM in standard buffer, prewarmed at 37°C.

#### Procedure

Maintain sterile conditions at all times during the assay.

1. Turn on the fluorescence microscope and set the wavelengths on *λ_ex_* at 490 nm and *λ_em_* at 520 nm.
  Note: Some components in the fluorescent microscope need to warm up. Therefore, turn on the microscope approximately 20 min in advance for stable measurements.
2. Wash one well twice with prewarmed standard buffer.
3. Preincubate the well with 250 µL standard buffer at 37°C for 10 min.
4. Aspirate the buffer and incubate with 250 µL 4 µM CDFDA in standard buffer at 37°C for 10 min.
5. Aspirate the CDFDA solution.
6. Make the room where the fluorescence microscope is located dark.
7. Focus on the cells using the light microscope and take images of the area of interest.
8. Switch off the light microscope lamp but not the fluorescence lamp without changing the plate position. If necessary, readjust the focus to make the bile canaliculi clearly visible and take images.

### Incubation of SCHH in presence or absence of the bile salt mixture

Incubation with test compound in presence and absence of bile salts is typically performed for 24 h. If longer incubations are required (*e.g.* 48 h), the medium is changed again after 24 h (day 6) with fresh medium containing the same initial concentrations of test compound and bile salts as 24 h before.

#### Materials

• Solution 1: culture medium with no compound and 0.2% DMSO. This solution should be filtered through a 0.2 µm filter and prewarmed for 30 min at 37°C.
• Solution 2: culture medium with following (50×) bile salts: 132 µM glycocheno-deoxycholic acid (GCDCA), 39 µM chenodeoxycholic acid (CDCA), 38 µM glycodeoxycholic acid (GDCA), 40 µM deoxycholic acid (DCA), and 35 µM glycocholic acid (GCA). The DMSO concentration should be adjusted to 0.2%. The solution medium should be filtered through a 0.2 µm filter and prewarmed for 30 min at 37°C.
• Solution 3: culture medium with *double concentrated* positive control: 30 µM cyclosporin A. The DMSO concentration should be adjusted to 0.2%. This solution should be filtered through a 0.2 µm filter and prewarmed for 30 min at 37°C.
• Solution 4: culture medium with *double concentrated* test compound. The DMSO concentration should be adjusted to 0.2%. This solution should be filtered through a 0.2 µm filter and prewarmed for 30 min at 37°C.
• Solution 5: control medium *with* bile salts, prepared by mixing solution 1 and solution 2 in a 1:1 ratio.
• Solution 6: culture medium with positive control *without* bile salts, prepared by mixing solution 1 and solution 3 in a 1:1 ratio.
• Solution 7: culture medium with positive control *with* bile salts, prepared by mixing solution 2 and solution 3 in a 1:1 ratio.
• Solution 8: culture medium with test compound *without* bile salts, prepared by mixing solution 1 and solution 4 in a 1:1 ratio.
• Solution 9: culture medium with test compound *with* bile salts, prepared by mixing solution 2 and solution 4 in a 1:1 ratio.

#### Procedure

1. On day 5, aspirate the medium and preincubate the cells with solution 1, solution 6 or solution 8 for 2 h.
  Note: this preincubation step is performed to enable the compounds to interact with the cellular machinery.
2. After 2 h, aspirate the medium and immediately incubate the cells with solution 1, solution 5, solution 6, solution 7, solution 8 or solution 9 for 22 h.

### Measurement of urea

The urea assay is performed on day 6 or day 7 for 24 h and 48 h incubations, respectively.

#### Materials

• Solutions for the color reagent. These solutions can be stored at 4°C for maximum 6 months.
  ○ Ferric chloride 1 mM solution in purified water.
  ○ Thiosemicarbazide 8 mM solution in purified water.
  ○ Diacetyl monoxime 119 mM solution in purified water.
  ○ Solution A prepared by slowly adding 30 mL of concentrated sulfuric acid and 10 mL of orthophosphoric acid to 60 mL of the FeCl_3_ solution and mixed gently.
    Note: perform this procedure on ice as this is an exothermic reaction. Wear appropriate protective gear.
  ○ Solution B prepared by mixing the thiosemicarbazide solution and the diacetyl monoxime in a 1:1 ratio.
• Incubation buffer: HBSS containing 10 mM HEPES, 2 mM glutamine, 10 mM ammonium chloride and 3 mM ornithine, filtered through a 0.2 µm filter and prewarmed at 37°C.
• 96-well thermostable plate with aluminum cover slip.

#### Procedure

1. Aspirate the culture medium.
2. Wash the wells twice with 250 µL prewarmed incubation buffer. Aspirate the remaining buffer.
3. Add 125 µL prewarmed incubation buffer to each well and keep the plate at 37°C for 1 h.
4. In the meantime, prepare the calibration curve in incubation buffer (range 5 µM–500 µM) and preheat a water bath to 85°C.
5. Prepare the color reagent by mixing solution A and B in a 2:1 ratio.
  Note: prepare and use the color reagent immediately.
6. After 1 h incubation, transfer 60 µL of the incubation buffer in every well of cells, blank and calibration curve to a 96-well thermostable plate.
7. Add 240 µL color reagent to each well.
8. Cover the plate with an aluminum cover slip to avoid evaporation.
9. Heat the plate with the incubation mixture to 85°C in a water bath for 20 min.
  Note: as heat is a crucial parameter for the reaction to occur, make sure the temperature is constant during the incubation.
10. Cool the plate down for 5 to 10 min at 4°C.
11. Transfer 250 µL of each well to a transparent 96-well plate and measure the absorbance at 525 nm and room temperature.

### Data analysis

1. Subtract the mean of the blank from all measured values.
2. Fit a calibration curve using linear regression.
3. Use the calibration curve to calculate the concentration of each condition.
4. For each condition, calculate the mean and standard deviation.
5. Calculate DICI ( ±  SD) according to following formula:
  $$DICI=\frac{urea\;formation_{test\;compound+bile\;salts}}{urea\;formation_{test\;compound}}$$
6. Calculate the SM according to following formula:
  $$SM=\frac{highest\;concentration\;with\;DICI\geq 0.78}{C_{max,\;total}\left(\mu M\right)}$$
  where *C*_max,total_ represents the mean total peak plasma concentration reported (or predicted) for humans.

## Method validation

### Optimization of bile salt mixture

The human bile salt pool is highly complex as it is composed of more than 50 different bile salts [13], [14], [15], [16], [17]. The most *in vivo*-relevant bile salt mixture would contain all these bile salt species at the same levels that they are found in human plasma. To simplify the *in vitro* assay, yet closely resembling the *in vivo* situation, we composed the bile salt mixture based on the following criteria: (1) the plasma levels of the selected bile salts had to be among the highest reported in humans, and (2) when used as a mixture in SCHH at their final concentrations no toxic effects should be observed.

**Fig. 2:**
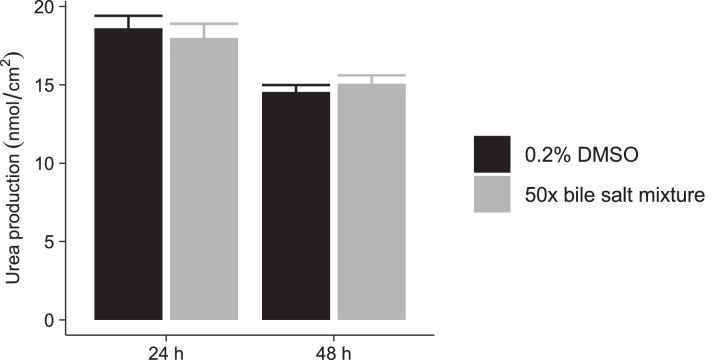
Urea production of sandwich-cultured human hepatocytes (mean ± SEM, *n* = 7 batches, measured in triplicate) after 24 h and 48 h of incubation with either 0.2% dimethylsulfoxide (DMSO) or a 50× concentrated bile salt mixture. Statistical significance was evaluated using a Student's *t*-test (*p*<0.05).

We selected GCDCA, GCA, DCA, GDCA and CDCA as they are the most abundant bile salt species in human plasma [14], [15], [16], [17] and because their plasma levels have been shown to undergo most pronounced increases during cholestasis [14,^17^,18]. The final concentration (50×) of the bile salt mixture was selected such that it would not affect the urea formation of the cultured cells. Fig. 2 illustrates the urea production of SCHH originating from seven different donors after 24 h and 48 h of incubation with either 0.2% DMSO or the 50× concentrated bile salt mixture. No significant difference between both groups after both 24 h and 48 h could be observed, indicating that the bile salt mixture does not affect the biochemical and functional integrity of the cultured cells.

### Choice of *in vitro* endpoint

During DIC, exposure to the drug in question primarily results from the pathophysiological accumulation of bile salts. In humans, bile salt-induced toxicity is predominantly characterized by increases in serum markers of necrotic, but not apoptotic, cell death [19]. Popular examples of necrosis assays include the lactate dehydrogenase (LDH) release assay and the (3-(4,5-dimethylthiazol-2-yl)-2,5-diphenyltetrazolium bromide (MTT) assay [20,21]. These assays are late markers of toxicity since they measure cell death rather than cell functionality. Our DIC assay however does not use a necrotic marker and aims to determine the hepatocyte functionality in presence of cholestatic drugs. Urea production from ammonia as the *in vitro* endpoint can be considered an early marker of (decreasing) cell functionality. Indeed, hepatocytes convert toxic ammonia to non-toxic urea through the urea cycle and a decrease in urea formation (compared to control conditions) is directly related to the loss of hepatocyte-specific function and biochemical and functional integrity [22].

### Evaluating assay performance using 24 test compounds

We used the data of the historical controls throughout all our experiments and calculated the mean 90% CI of the DICI value. For 24 h and 48 h incubation, the mean 90% CI were 0.78–1.22 and 0.78–1.21, respectively. The lower boundary of the 90% CI was used to define a data variability-driven cut-off value of 0.78 for both 24 h and 48 h incubation. This value remains consistent with our previously used arbitrary cut-off value of 0.80.

The performance of the DIC assay was evaluated by exposing several batches of SCHH to a set of 24 test compounds at various concentrations [6,7]. The selected test compounds spanned a range of physicochemical properties as well as mechanisms of toxicity including mitochondrial toxicity, formation of reactive metabolites, inhibition of hepatobiliary transporters and immune responses [23]. The test compounds were subdivided into three groups based on their mechanism of toxicity. Compounds in group A are compounds that induced liver injury without causing cholestasis (*i.e.,* acetaminophen, amiodarone, diclofenac, tolcapone, ximelagatran, perhexiline, fialuridine and warfarin). Compounds in group B are cholestatic compounds (*i.e.,* troglitazone, nefazodone, bosentan, cyclosporin A, midecamycin, troleandomycin, erythromycin, rosiglitazone, ticlopidine, chlorpromazine, ritonavir and atazanavir). Compounds in group C were considered as negative controls and are not reported to cause significant liver injury in humans (*i.e.,* entacapone, metformin, buspirone and pioglitazone).

A DICI value was determined for all test compounds after 24 h of exposure and 48 h of exposure. Table 1 summarizes the obtained DICI values of all test compounds. Values in bold are considered cholestatic based on the arbitrary cut-off value of 0.80, whereas underscored values are deemed to be cholestatic based on the new cut-off value of 0.78. Table 2 summarizes the calculated specificity and sensitivity using the two cut-off values. Although the sensitivity of both cut-offs remained unchanged for both incubation times (*i.e.* 58% and 71%), the specificity significantly increased when applying the 0.78 cut-off value.

**Table 1:** Mean (± SD) drug-induced cholestasis index (DICI) values of the 24 test compounds after 24 h of exposure and after 48 h of exposure in sandwich-cultured human hepatocytes. Values in bold and underscored show a disturbance in bile salt homeostasis according to the arbitrary cut-off value of 0.80 and based on the new cut-off value of 0.78, respectively. N.D.; not determined.

Class	Compound	Concentration (µM)	DICI 24 h		DICI 48 h	
			Mean	SD	Mean	SD
A	Acetaminophen	200	0.92	0.07	0.96	0.11
		500	0.91	0.09	1.02	0.07
		1000	0.95	0.24	1.02	0.01
	Amiodarone	5	0.97	0.02	1.03	0.17
		10	1.09	0.16	1.10	0.16
		20	1.24	0.05	0.99	0.06
		25	0.96	0.16	0.95	0.20
	Diclofenac	100	1.18	0.16	1.19	0.23
		300	1.34	0.3	1.04	0.19
		500	1.16	0.24	1.00	0.19
	Tolcapone	5	0.98	0.18	1.01	0.06
		10	1.05	0.16	0.89	0.24
		50	0.91	0.23	**0.79**	0.22
	Ximelagatran	25	0.85	0.17	0.95	0.21
		50	0.95	0.14	0.93	0.09
		150	1.02	0.07	1.05	0.09
	Perhexiline	3	1.00	0.11	**0.80**	0.05
		9	1.03	0.18	0.98	0.30
		65	1.10	1.49	1.02	0.07
	Fialuridine	10	0.88	0.31	0.92	0.16
		20	0.94	0.35	**0.79**	0.16
		30	0.95	0.39	1.26	0.64
		50	**0.79**	0.17	1.19	0.43
		100	1.12	0.20	0.89	0.27
		300	0.82	0.21	0.96	0.11
	Warfarin	500	1.31	0.19	N.D.	N.D.
B	Troglitazone	20	0.98	0.09	1.09	0.10
		50	1.02	0.23	1.00	0.10
		75	**0.78**	0.13	**0.79**	0.07
		100	**0.74**	0.09	**0.24**	0.16
	Nefazodone	10	**0.69**	0.11	1.18	0.37
		30	**0.77**	0.15	**0.37**	0.15
		130	1.09	0.87	4.80	3.72
	Bosentan	50	1.07	0.10	1.06	0.13
		100	0.99	0.13	0.87	0.14
		200	1.15	0.19	1.02	0.24
	Ritonavir	1	0.95	0.09	0.92	0.12
		20	0.87	0.08	1.01	0.16
		50	**0.55**	0.21	**0.79**	0.14
		100	**0.66**	0.06	**0.40**	0.28
	Cyclosporin A	15	**0.70**	0.17	**0.62**	0.15
	Midecamycin	100	**0.71**	0.16	1.07	0.07
	Troleandomycin	10	1.00	0.09	0.83	0.26
		12.5	1.41	0.48	N.D.	N.D.
		20	1.12	0.13	0.91	0.08
		25	1.13	0.11	N.D.	N.D.
		50	0.93	0.14	N.D.	N.D.
		100	0.97	0.15	N.D.	N.D.
	Erythromycin	50	0.94	0.21	N.D.	N.D.
		100	1.29	0.30	N.D.	N.D.
		200	1.42	0.33	N.D.	N.D.
	Rosiglitazone	50	1.08	0.19	N.D.	N.D.
		100	1.27	0.13	N.D.	N.D.
	Ticlopidine	100	**0.51**	0.22	N.D.	N.D.
	Chlorpromazine	10	**0.48**	0.09	N.D.	N.D.
	Atazanavir	50	0.87	0.11	0.86	0.17
		150	1.19	0.37	**0.73**	0.09
C	Entacapone	10	1.11	0.19	1.02	0.33
		30	1.07	0.11	0.98	0.11
		130	**0.62**	0.07	**0.10**	0.03
	Metformin	150	1.09	0.30	1.34	0.63
		300	1.03	0.30	1.26	0.62
		900	1.12	0.42	1.18	0.49
	Buspirone	0.5	**0.69**	0.50	0.82	0.27
		1	0.87	0.69	0.90	0.36
		5	**0.74**	0.53	0.95	0.39
		10	0.82	0.36	0.95	0.26
		25	0.84	0.29	**0.71**	0.20
		50	**0.68**	0.06	**0.48**	0.21
		150	**0.53**	0.02	**0.37**	0.09
	Pioglitazone	50	1.15	0.09	1.06	0.14
		100	1.00	0.04	0.88	0.05
		300	0.94	0.06	0.98	0.06

**Table 2:** Sensitivity and specificity of the previously used cut-off value of 0.80 and the new cut-off value of 0.78 after 24 h and 48 h of incubation. Sensitivity was calculated as the ratio of flagged class B compounds (true positives) and the sum of flagged class B compounds and unflagged class B compounds (false negatives). Specificity was calculated as the ratio of unflagged class C compounds (true negatives) and the sum of unflagged class C compounds and flagged class A and C compounds (false positives).

	Cut-off value 0.80		Cut-off value 0.78	
	24 h	48 h	24 h	48 h
Sensitivity	58%	71%	58%	71%
Specificity	40%	29%	50%	50%

To translate the *in vitro* cholestasis risk of a test compound to the *in vivo* situation, we used the safety margin (SM) concept. In our previously published protocol, the SM reflected the ratio between the lowest *in vitro* concentration of the tested compound that yielded a DICI-positive result (*i.e.,* the LOAEL) and the compound's *C*_max,total_. However, during non-clinical risk assessment the NOAEL is a widely used approach for safety evaluations supported by regulatory agencies [9]. Taking this consideration into account, we hereby propose to calculate the SM as the ratio between the highest *in vitro* concentration of the tested compound that yielded a DICI-negative result (*i.e.,* the NOAEL) and the compound's *C*_max,total_. For compounds with DICI values below 0.78 at all concentrations tested, the lowest concentration was used to calculate a maximum SM. Table 3 summarizes the SMs, calculated based on the new DICI cut-off value of 0.78, after 24 and 48 h incubation determined by either the LOAEL or NOAEL method. Values in bold indicate differences between the original and modified calculation methods. At 24 h incubation, the SMs of troglitazone, ritonavir, nefazodone, entacapone and buspirone as calculated by the NOAEL method, were lower as compared to the LOAEL method, whereas the SMs of other compounds remained unchanged between both methods. At 48 h incubation, the SM of fialuridine was higher whereas the SMs of troglitazone, nefazodone, bosentan, ritonavir, entacapone, buspirone and atazanavir were lower as compared to the 24 h SM values. Not unexpectedly, this indicates that the NOAEL method is more sensitive towards detecting potentially cholestatic compounds as compared to the LOAEL method.

**Table 3:** Safety margins (SMs) of the 23 test compounds calculated either as the ratio between the lowest *in vitro* concentration that yielded a DICI-positive result (< 0.78) and the total plasma concentration (*C*_max,total_) of the compound (*i.e.,* the LOAEL method), or as the ratio between the highest *in vitro* concentration that yielded a DICI-negative result (≥ 0.78) and the *C*_max,total_ of the compound (*i.e.,* the NOAEL method). The SMs were calculated based on the new DICI cut-off value of 0.78. Values in bold differ between both methods. N.D.; not determined.

Class	Compound	*C*_max,total_ (µM)	DICI-based SM obtained with the NOAEL method		DICI-based SM obtained with the ‘original’ LOAEL method		References
			24 h	48 h	24 h	48 h	
A	Acetaminophen	139	7.19	7.19	7.19	7.19	[24]
	Amiodarone	0.81	30.86	30.86	30.86	30.86	[24,25]
	Diclofenac	7.99	62.58	62.58	62.58	62.58	[24,25]
	Tolcapone	47.6	1.05	1.05	1.05	1.05	[3]
	Ximelagatran	0.3	500	500	500	500	[26, 27]
	Perhexiline	2.16	30.09	30.09	30.09	30.09	[24]
	Fialuridine	0.64	468.75	**468.75**	468.75	**46.88**	[3]
	Warfarin	6.97	71.74	N.D.	71.74	N.D.	[28]
B	Troglitazone	6.39	**7.82**	**11.74**	**15.65**	**15.65**	[24,25]
	Nefazodone	4.26	**2.35**	**2.35**	**30.52**	**7.04**	[3]
	Bosentan	7.39	27.06	**6.77**	27.06	**27.06**	[3]
	Ritonavir	13.24	**1.51**	**1.51**	**7.55**	**7.55**	[29], [30], [31]
	Cyclosporin A	0.77	19.48	19.48	19.48	19.48	[3]
	Midecamycin	2	50	50	50	50	[32]
	Erythromycin	3.97	50.38	N.D.	50.38	N.D.	[33,34]
	Rosiglitazone	1.776	56.31	N.D.	56.31	N.D.	[35]
	Ticlopidine	2.898	34.51	N.D.	34.51	N.D.	[36,37]
	Chlorpromazine	0.0376	265.96	N.D.	265.96	N.D.	[38,39]
	Atazanavir	4.82	31.12	**10.37**	31.12	**31.12**	[40], [41], [42]
C	Entacapone	4.34	**6.91**	**6.91**	**29.95**	**29.95**	[43]
	Metformin	7.75	116.13	116.13	116.13	116.13	[44]
	Buspirone	0.01	**50**	**1000**	**5000**	**15000**	[3]
	Pioglitazone	2.95	101.69	101.69	101.69	101.69	[24,25]

**Fig. 3:**
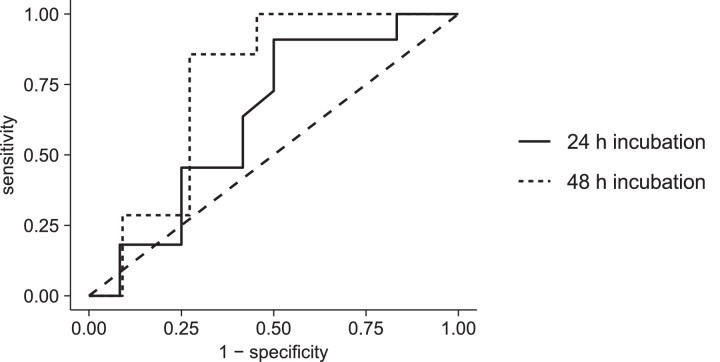
ROC curves for different cut-off values of the SM to predict DIC after 24 h and 48 h incubation. The area under the ROC curve was 0.633 (90% CI 0.430–0.836) for 24 h incubation and 0.753 (90% CI 0.553–0.954) for 48 h incubation. The dashed line represents the reference line for classification by random chance.

We previously maintained a cut-off value of 30 for the SM, based on a study conducted by Yao et al. [10]. However, this value was never verified for DIC detection in our assay. In the current protocol we have reassessed all our generated data and performed a ROC analysis using the pROC package in R version 3.6.2 [45]. SMs were calculated using the NOAEL-based DICI value as described above. For troleandomycin no *C*_max,total_ was available and no SM could be calculated. This compound was therefore excluded for analysis. For warfarin, erythromycin, rosiglitazone, ticlopidine and chlorpromazine, the DICI value was only determined after 24 h incubation. The resulting ROC curves are depicted in Fig. 3. The area under the ROC curve was 0.633 (90% CI 0.430–0.836) for 24 h incubation (*n* = 23) and 0.753 (90% CI 0.553–0.954) for 48 h incubation (*n* = 18). There was no statistically significant difference between the AUC of both curves (*p* = 0.491). The optimal cut-off values for the SM were determined based on the closest point to the top left corner [46]. For the 24 h incubation, the optimal SM value amounted to 59.44 with a corresponding sensitivity and specificity of 91% and 50%, respectively. For the 48 h incubation, the optimal SM value amounted to 28.58 with a corresponding sensitivity and specificity of 86% and 73%, respectively. The sensitivity and specificity of the previously used SM of 30 were 45% and 75% after 24 h incubation and 86% and 73% after 48 h incubation, respectively.

## Discussion

In this modified protocol, we describe a non-destructive assay for *in vitro* hepatocyte-based prediction of drug-induced cholestasis. The *in vitro* assay is based on quantifying changes in the ability of primary hepatocytes to convert toxic ammonia to non-toxic urea when co-exposed to a test compound and bile salts.

In order to quantify the *in vitro* cholestasis potential of a given test compound within a range of concentrations, concentration dependent DICI profiles are generated. The DICI reflects the ratio in urea production of SCHH co-exposed to a test compound and bile salts or to the test compound alone. In our previously published protocol [6], [7], [8], compounds with a DICI value lower than 0.80 were considered cholestatic. This cut-off value was chosen arbitrarily and had no statistical basis. In the current protocol, we propose a modified approach that is relying on the observed variability in DICI values under control conditions. The new cut-off value of 0.78 was defined as the lower boundary of the 90% CI of the DICI values calculated based on the historical controls throughout all our experiments. Our data have shown that the new cut-off value yields the same sensitivity, but higher specificity. In other words, less compounds will falsely be classified as positive at the same rate of identifying true positives. Therefore, and because the new cut-off value takes the variability of the controls into account, we consider this approach to be an improvement to the previously used value of 0.80.

In order to translate the obtained *in vitro* DICI values to the *in vivo* situation, we had previously applied the concept of a SM. This SM was based on the work of Yao et al. who investigated the risk of drugs prolonging the QT interval. In this study, they defined a SM of 30 which was able to distinguish between drugs causing torsade de pointes, a life-threatening condition, and those who were not [10]. In the current protocol, we have proposed to calculate the SM as the ratio between the NOAEL and the test compound's *C*_max,total_, rather than the ratio between the LOAEL and the test compound's *C*_max,total_, as in our original protocol. A direct comparison between both approaches indicated that the NOAEL method is more specific as compared to the LOAEL method (see Table 2). We subsequently performed a ROC analysis to determine the cut-off values for the SM. At 24 h incubation, the cut-off value was 59.44. This was remarkably higher than the previously used value of 30 based on the work of Yao et al. [10]. However, the area under the ROC curve for 24 h incubation was only 0.627 indicating a poor accuracy of the assay. Indeed, with a sensitivity of 90% and specificity of 46% for that cut-off value, most of the DIC causing compounds are identified but many others are falsely identified as cholestatic as well. After 48 h incubation, on the other hand, we determined a cut-off value of 28.58 with a slightly lower sensitivity of 83% but higher specificity of 67% as compared to 24 h incubation. This value is also close to the earlier used value of 30, thereby validating the previously used SM. In addition, the analysis showed good accuracy at 48 h incubation, demonstrated by an area under the ROC curve of 0.764.

Based on the improved specificity of the NOAEL method and the SM determined with ROC analysis, we propose to incubate all compounds for 48 h. Although the sensitivity as well as the specificity are better at 24 h incubation for both methods, the ROC analysis showed that 48 h incubation allows better prediction of the cholestatic risk *in vivo*. Furthermore, we believe that using the NOAEL to calculate the DICI value is more justified and will improve safety as it is more conservative than using the LOAEL.

Currently, both the DICI values and SMs are calculated based on the total concentration of the drug present in the *in vitro* system and/or in plasma (*C*_max,total_). However, it is generally accepted that only the fraction of the drug that is freely available may exert toxic effects [47]. Consequently, determination of the DICI values and SMs should be based on both unbound *in vitro* and/or *in vivo* concentrations of the drug in question. However, for highly bound compounds it is difficult to predict the fraction unbound in plasma (*f*_u,p_) accurately. To account for *in vivo* relevant protein binding, the culture medium can be supplemented with 4% bovine serum albumin (BSA). This mimics the *in vivo* protein binding and avoids inaccurate calculations without the need for additional experimentation. Alternatively, the unbound tissue partition coefficient (Kp_u,u_) can be used, defined as the ratio between the unbound intracellular liver concentration and the unbound concentration in the plasma [48]. In the latter case, also the actual *in vitro* intracellular unbound concentration should preferably be determined and used for calculation of the SM.

Our assay can be used for the detection of DIC in early stages of the drug development process. Even for compounds for which no clinical data is available a SM can be estimated based on predicted *C*_max,total_ values from physiologically-based pharmacokinetic (PBPK) modeling. Especially when sufficient preclinical data is available, such models have shown to provide valuable estimates of clinical exposure [49].

Due to the limited culture time of SCHH and decreasing expression and activity of transporters and metabolizing enzymes, the described methodology is currently not applicable to evaluate long-term toxicity effects [50]. However, the urea assay could further be optimized for *in vitro* models enabling longer culture times (*e.g.* 3D spheroids). It was previously shown that 3D spheroids from primary human hepatocytes show relevant expression of bile salt transporters such as MRP2 and BSEP and are suitable for detection of cholestasis through measurements of intracellular bile salt accumulation after prolonged exposure [51]. Similarly, our method could be applied to other advanced *in vitro* culture systems that include co-culture of hepatocytes with Kupffer cells. Recent studies with rat and pig hepatocytes have shown that this *in vitro* model is suitable for investigation of immune-mediated toxicity mechanisms [52,53], which have been shown to also play a role in the development of cholestasis. This is also relevant in the context of drug-induced cholestasis. Bile salts have indeed been reported to induce the expression of proinflammatory cytokines in mouse hepatocytes, and for instance the C–C motif chemokine ligand 2 (CCL2) levels are also elevated in cholestatic patients [54]. In addition, in-house data suggests that certain bile salts may act as early markers of cholestasis development as well. These new insights might open opportunities for additional early markers of cholestasis.

In conclusion, we described an optimized protocol for an assay to determine the cholestatic potential of test compounds based on sandwich-cultured human hepatocytes. We determined a data-driven cut-off for the drug-induced cholestasis index (DICI) value that is based on the observed variability across our historical control data. Subsequently, we also modified our algorithm for predicting a safety margin, *i.e.* by relying on the DICI-based NOAEL rather than LOAEL in the original method. Importantly, the SM allows proper translation of the highest *in vitro* concentration devoid of cholestasis potential to an *in vivo* risk for cholestasis development. These analyses showed that incubation of the test compounds for 48 h results in reliable DIC assessment. The previously used SM cut-off of about 30 was confirmed by a ROC analysis. The methodology described here is a valuable tool to detect the cholestasic potential of drug candidates during early drug development.

## Declaration of Competing Interest

The authors declare that they have no known competing financial interests or personal relationships that could have appeared to influence the work reported in this paper.
